# Supplementary material for: Vancomycin Penetration in Brain Extracellular Fluid of Patients with Post-Surgical Central Nervous System Infections: An Exploratory Study
Source: Medicina (Kaunas). 2025 Nov 5;61(11):1989. doi: 10.3390/medicina61111989 (PMC12654646; doi:10.3390/medicina61111989)
Supplement: Supplementary file 1 [file medicina-61-01989-s001.zip › medicina-3871168-supplementary.pdf]

Table S1a. Demographics, baseline clinical characteristics

Patient No.	Age (years)	Sex	BMI (kg/m²)	ClCr (mL/min)	Renal Function Change	Liver Function	CRP (mg/L)	ANC ($x10^9/L$)	CSF protein (g/L)	GCS score	Underlying Disease
**1**	28	Male	29.4	146	No	Normal	58	5	7	6-7	Frontobasal encephalocele
**2**	69	Male	22.1	92	Yes (decrease)	Normal	118	22	88	10	Traumatic brain injury with subdural haemorrhage
**3**	74	Male	24.7	82	No	Normal	116	16	-	5	Hemorrhagic stroke: intracerebral & intraventricular hematoma
**4**	40	Female	25.1	271	No	Normal	131	12	5	7	Arteriovenous malformation with secondary intracerebral & intraventricular hemorrhage after embolization
**5**	53	Male	24.9	183	No	Normal	55	13	8	10	Ruptured aneurysm with subarachnoid hemorrhage

BMI: body mass index; ClCr: creatinine clearance; GCS: Glasgow coma scale; CRP: C-reactive protein; ANC: absolute neutrophil count; CSF: cerebrospinal fluid

Table S1b. Treatment details and microbiology

Patient No.	Vancomycin Dose (mg/kg/24h)	Infusion Type	Intraventricular Dose	Other Antibiotics	CSF Culture
**1**	22	Intermittent	20 mg daily	Ceftriaxone	Negative
**2**	10	Intermittent	None	Ceftriaxone	*Proteus mirabilis, Escherichia coli, Klebsiella pneumoniae*
**3**	25	Continuous	None	Ceftazidime	Negative
**4**	36	Continuous	10 mg daily	Meropenem	*Staphylococcus capitis*
**5**	28	Continuous	None	Meropenem	Negative

CSF: cerebrospinal fluid

Table S1c. Neuroinfection markers, ICU/hospital stay, and clinical outcomes

Patient No.	Cerebral Edema	Perifocal Edema	Increased ICP	Outcome	ICU Stay (days)	Hospital Stay (days)	Vancomycin Duration (days)	CSF Lactate >3.5	CSF/Blood Glucose Ratio <0.4	Cell Index >5	Fever	Other Notes
1	No	Yes	Data unavailable, clinically unsuspected	Cure	4	112	39	Yes	Yes	No	Febrile	N/A
2	No	No	Data unavailable, clinically unsuspected	Death	8	36	4	Yes	Yes	Yes	Afebrile	N/A
3	No	Yes	Data unavailable, clinically unsuspected	Cure	5	35	16	Data not available	Data not available	Yes	Afebrile	N/A
4	No	Yes	Data unavailable, clinically unsuspected	Cure	8	71	33	Yes	Yes	Yes	Febrile	N/A
5	No	No	No	Cure	5	31	12	Yes	Yes	No	Subfebrile	Later neuroinfection excluded

ICP: intracerebral pressure; ICU: intensive care unit; CSF: cerebrospinal fluid; N/A: not applicable

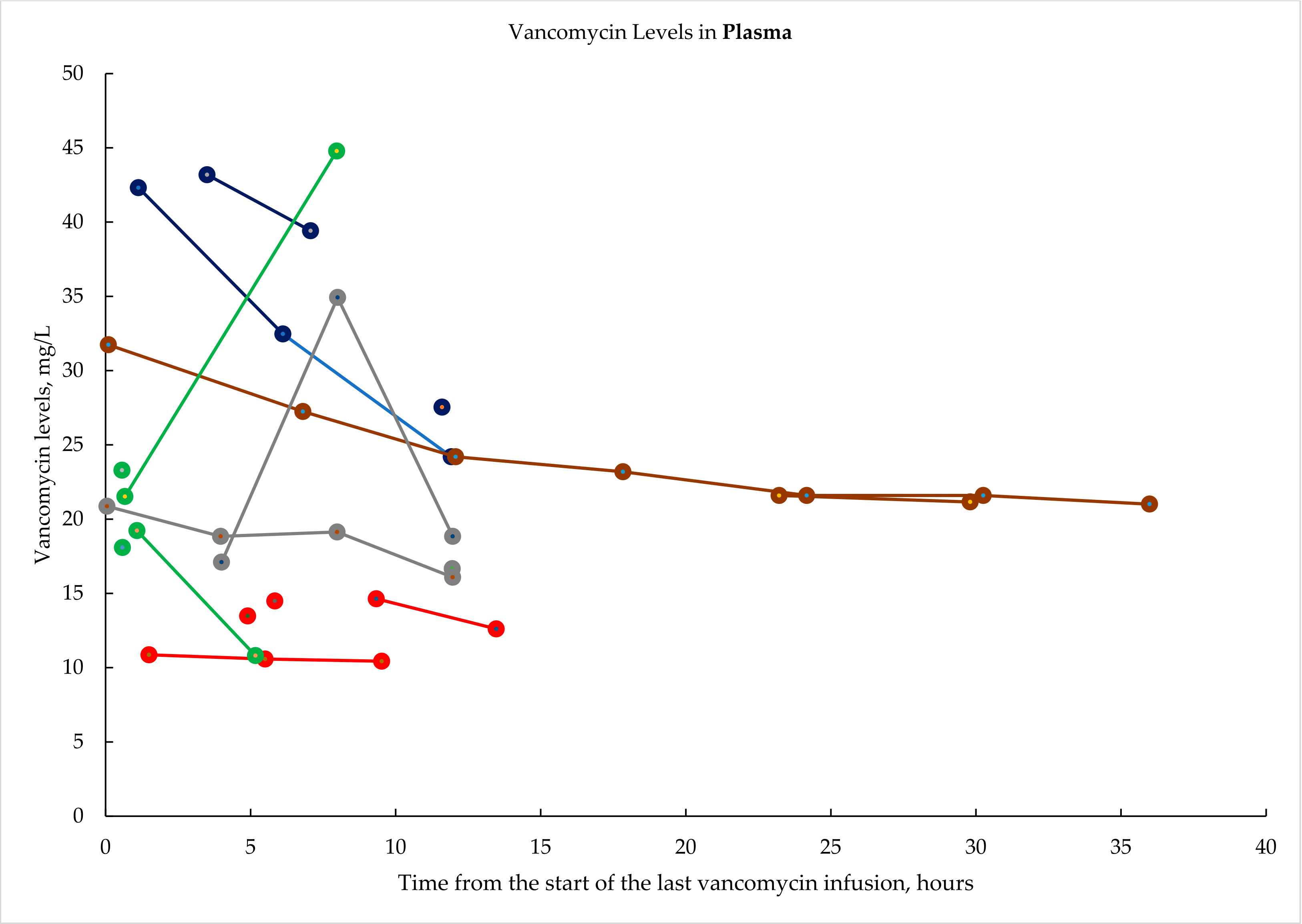

Figure S1. Vancomycin plasma concentrations plotted over the time curve. Different colors represent individual patients. Connected points indicate samples collected within the same dosing interval, whereas isolated points reflect single samples obtained for that interval.

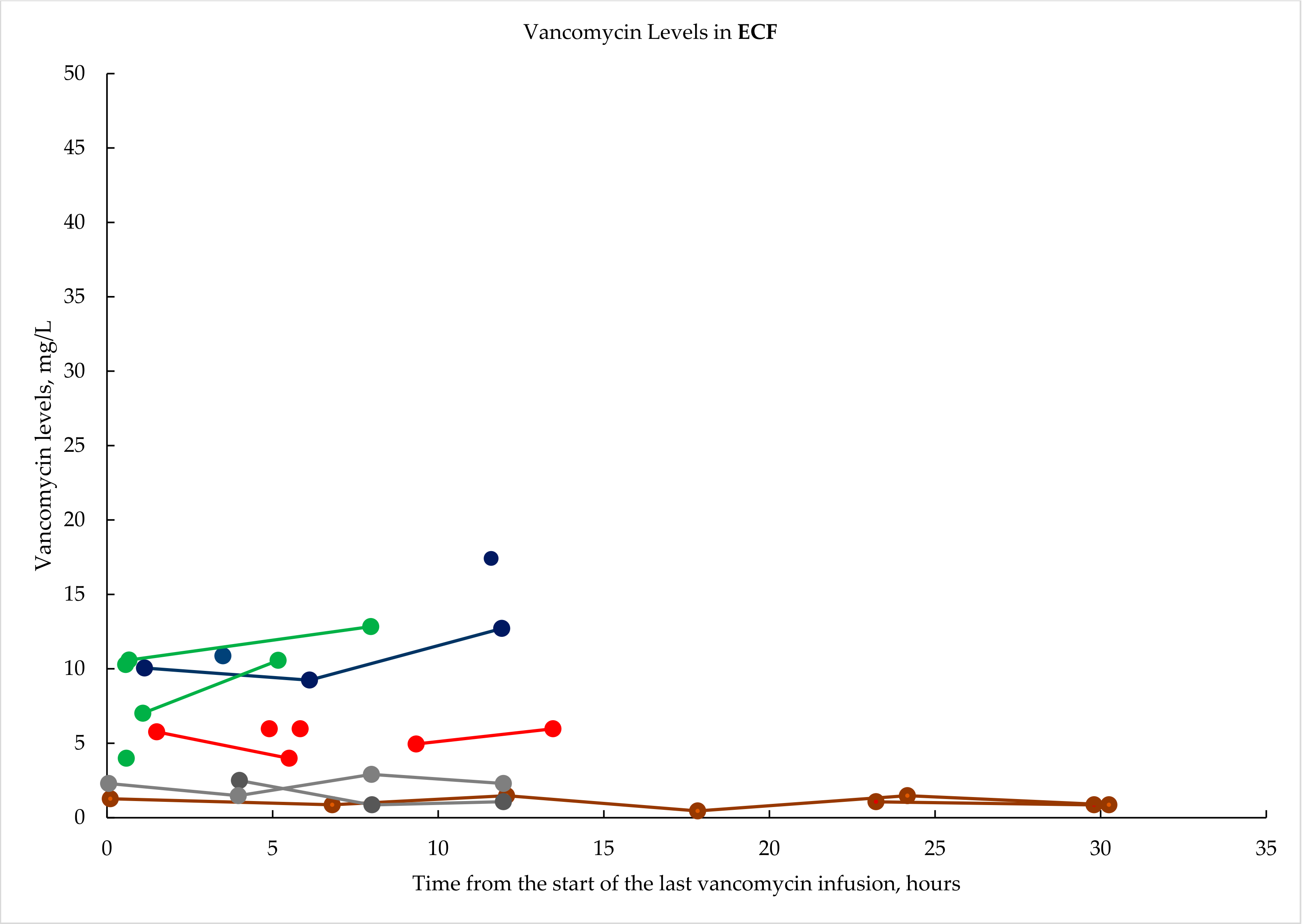

Figure S2. Vancomycin concentrations in brain ECF plotted over the time curve. Different colors represent individual patients. Connected points indicate samples collected within the same dosing interval. Unlike plasma, each ECF data point reflects an average concentration over a defined microdialysis collection period (typically 4-6 hours), rather than a single time-point measurement.

Calculation of Time to Steady State (tSS) [1]

The time to reach steady state (tSS) was estimated as five elimination half-lives ($t_{½}$), following the general pharmacokinetic principle:

- $tSS = 5 \times t_{½}$

The elimination half-life ($t_{½}$) was calculated using the following equation:

- $t_{½} = \frac{0.693 \times V_d}{CL_{vanco}}$

The volume of distribution (Vd) was considered 0.7 L/kg, representing the average value based on published literature.

Vancomycin clearance (CL_{vanco}) was estimated using the following relationship based on creatinine clearance (CLcr):

- $CL_{vanco} = (CLcr \times 0.689) + 3.66$

In patients with renal hyperfiltration, creatinine clearance values were capped at 100 mL/min to avoid underestimation of half-life. The calculated time to steady state (tSS) was then used to determine the initiation of microdialysis sampling. Sampling began at least one full dosing interval after the estimated steady-state time had elapsed to ensure pharmacokinetic equilibrium.

References:

1. Beringer, P.M. *Winter's Basic Clinical Pharmacokinetics, 6e*; Lippincott Williams & Wilkins, a Wolters Kluwer bu, 2018; pp. 467-496
